# Oligomerization of 2-chloroallyl alcohol by 2-pyridinecarboxylate complex of chromium(III) - new highly active and selective catalyst

**DOI:** 10.1038/s41598-018-26973-6

**Published:** 2018-06-05

**Authors:** Joanna Drzeżdżon, Artur Sikorski, Lech Chmurzyński, Dagmara Jacewicz

**Affiliations:** Faculty of Chemistry, University of Gdańsk, Wita Stwosza 63, 80–308 Gdańsk, Poland

## Abstract

The new 2-pyridinecarboxylate (2-pic) complex of chromium(III) has been designed and synthesized as a new highly active and selective oligomerization catalyst. The crystal structure of the new compound has been determined by X-ray diffraction. The composition and purity of [Cr(2-pic)_2_(OH_2_)_2_]NO_3_ have been confirmed by several spectroscopic methods and the elemental analysis. Furthermore, the new complex has been investigated towards its catalytic activity for the oligomerization of 2-chloro-2-propen-1-ol under the atmospheric pressure and at room temperature. It has turned out that the novel catalyst exhibits a very high catalytic activity. Consequently, [Cr(2-pic)_2_(OH_2_)_2_]NO_3_ belongs to a new generation of non-metallocene catalysts.

## Introduction

The organometallic complexes of chromium(III) are known as catalysts for the olefin polymerization^1^. The catalytic activity of the chromium(III) complexes is induced by the addition of methylaluminoxane (MAO) or its modified form (MMAO)^2^. The metallocene chromium(III) complexes exhibit a very high catalytic activity but unfortunately they are unstable under an industrial polymerization of olefins and their derivatives conditions^3,4^. Additionally, the decomposition of the metallocene complexes of chromium(III) is observed after their reaction with MAO.

Owing to the interesting catalytic activity of the non-metallocene complexes of chromium(III), these complexes are considered as a new generation of catalysts for olefins and their derivatives polymerization^5^. The example of the non-metallocene complex with a catalytic activity for ethylene polymerization is Cr[N(SiMe_3_)_2_]_2_I_2_ (43 g∙mmol^−1^∙h^−1^∙bar^−1^)^6^. This chromium(III) complex containing neutral ligands exhibits low catalytic properties. However, the non-metallocene catalysts include the chromium(III) complexes with monoanionic ligands a with moderate or high catalytic activity e.g. the bis(phosphino)amide complex of chromium(III) with the activity equal 500 g∙mmol^−1^∙h^−1^∙bar^−1 ^^7,8^, the chromium(III) complex with triptycenyl and 2-pyridylmethyl exhibits the 6970 g∙mmol^−1^∙h^−1^∙bar^−1^ catalytic activity^1^. The chromium(III) complexes with pyrrole–imino-amine/ether pro-ligands {ENN^R^}H (E = NH, R = H, 1a; E = NH, R = tBu, 1b; E = O, R = H, 1c) have a very high catalytic activity for the ethylene polymerization^9^. Thus, the non-metallocene chromium(III) complexes with monoanionic ligands are promising catalysts for the industrial olefin polymerization^10,11^.

Polyvinyl alcohol is used to produce hydrogels, to drugs production in the pharmaceutical industry and as a stabilizer in emulsions^12,13^. 2-Chloro-2-propen-1-ol is the derivative of vinyl alcohol (hydroxyethene). Thus, the product of the polymerization of this monomer (2-chloro-2-propen-1-ol) may exhibit similar properties and applications as polyvinyl alcohol. Therefore, 2-methyl-2-propen-1-ol as the derivative of 2-chloro-2-propen-1-ol was investigated as the substrate in production of renewable hydrogels by oligomerization^14^. In the literature, there is lack of information about the polymerization of 2-chloro-2-propen-1-ol using complex compounds as the catalysts. Recently, the results of our studies on the new type of chromium(III) catalysts (dipicolinate complexes of Cr(III) with 2,2′-bipyridine and its derivative as ligands) containing both organic cations and anions designed for the polymerization of 2-chloro-2-propen-1-ol has been published^15^. These new catalysts exhibit a very high catalytic activity. It has to be mentioned that the poly(2-chloroallyl alcohol) is prepared at the atmospheric pressure and the room temperature (21 °C)^15^.

This report is a continuation of our previous studies. We describe the structure of the new [Cr(2-pic)_2_(OH_2_)_2_]NO_3_ complex compound where 2-pic denotes the 2-pyridinecarboxylate anion. Moreover, the catalytic activity of the 2-pyridinecarboxylate complex of chromium(III) has been studied in the case of the 2-chloroallyl alcohol oligomerization. Furthermore, the catalytic activity of the novel catalyst has been compared with other known chromium(III)-based catalysts.

## Results

### The structure of the new complex

**Figure 1:**
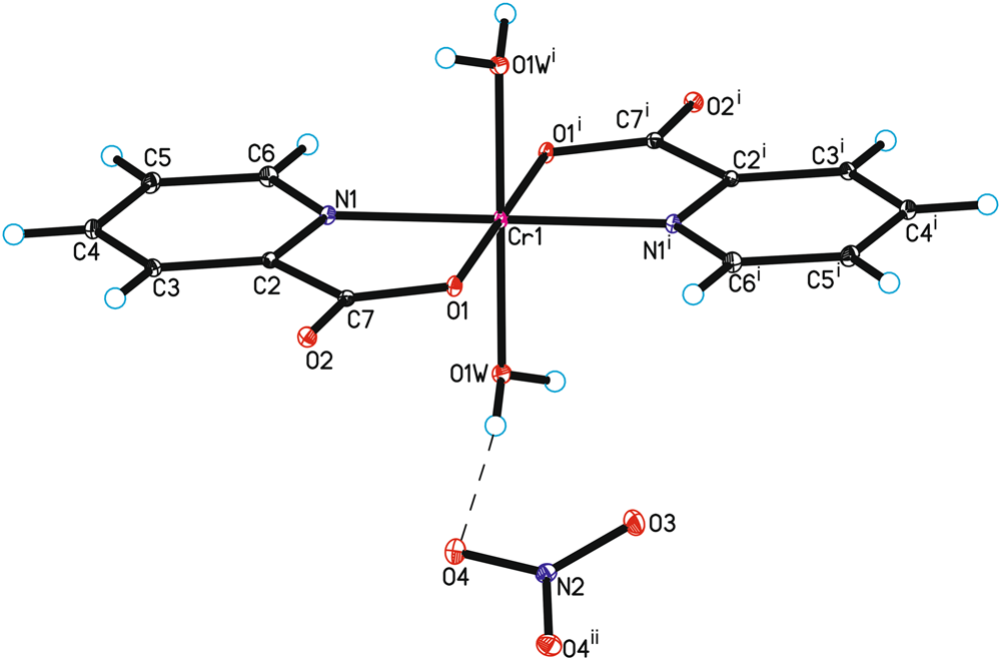
The molecular structure of [Cr(2-pic)_2_(OH_2_)_2_]NO_3_. Displacement ellipsoids are drawn at the 50% probability level (Symmetry codes: (i) −x + 1, −y, −z + 1 (ii) −x + 1, y, −z + ½).

The crystal structure of the novel chromium(III) complex –[Cr(2-pic)_2_(OH_2_)_2_]NO_3_ has been studied by the X-ray diffraction method. The molecular structure of the new complex has been shown in Fig. 1. The crystallographic data for [Cr(2-pic)_2_(OH_2_)_2_]NO_3_ have been collected in Supplementary Information. In the crystal structure of the [Cr(2-pic)_2_(OH_2_)_2_]^+^ a cation is arranged around a center of a symmetry, and in NO_3_^−^ anion N2 and O3 atoms lying on the rotational 2-fold axis (Fig. 1).

**Figure 2:**
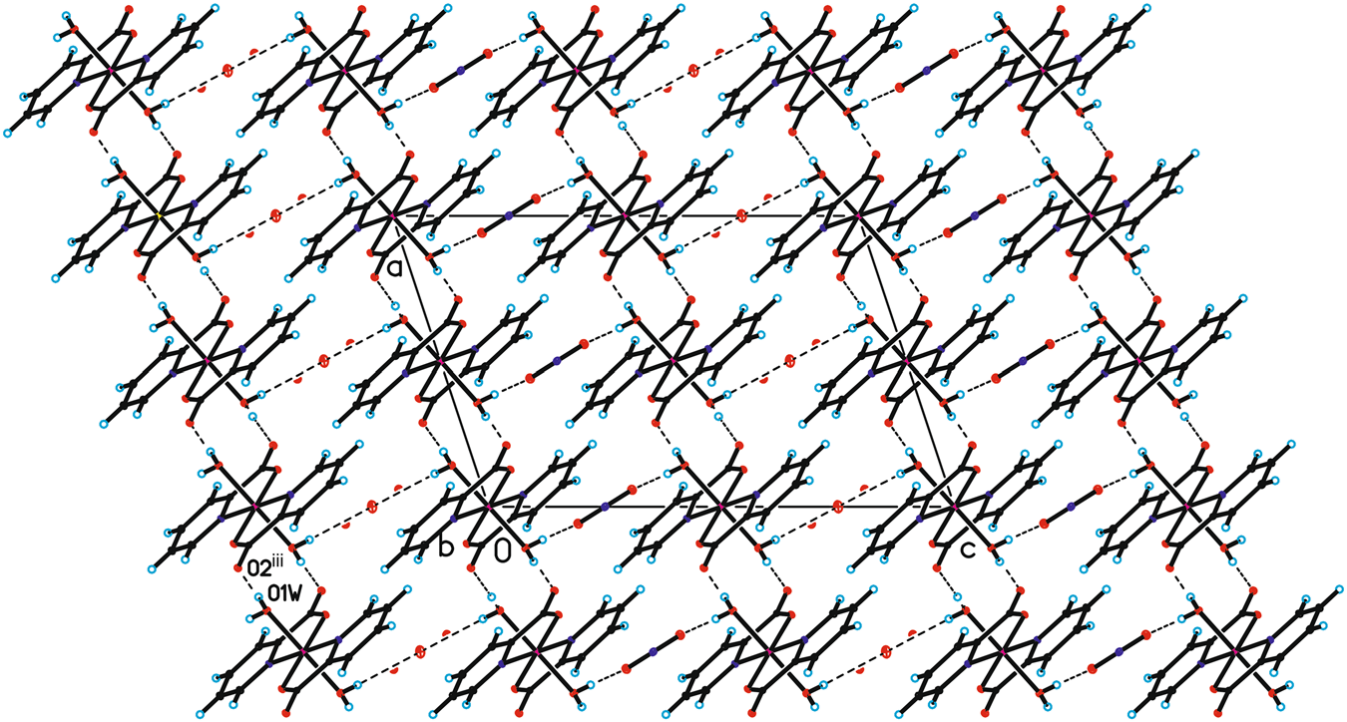
The crystal packing of the title compound viewed along the *b*-axis (hydrogen bonds and halogen bonds are represented by dashed lines; symmetry codes (iii)) ½ + x, −½ + y, z.

The geometric parameters (bond lengths and angles) characterizing both [Cr(2-pic)_2_(OH_2_)_2_]^+^ and in NO_3_^−^ are typical for these units (see Supplementary Information). In the crystal packing of tile compounds [Cr(2-pic)_2_(OH_2_)_2_]^+^ cations are linked via O1W–H1WA···O2 hydrogen bonds to form tapes along the [110] direction. The neighboring tapes are linked by O–H···O hydrogen bonds between water molecules and NO_3_^−^ anion forming a three-dimensional framework structure (Fig. 2).

The purity and composition of [Cr(2-pic)_2_(OH_2_)_2_]NO_3_ was confirmed by the elemental analysis. This test was conducted on the CARBO ERBA - O 1108 automated analyzer. Anal. Calcd for [Cr(2-pic)_2_(OH_2_)_2_]NO_3_ (%): C, 36.54, H, 3.07, N, 10.67. Found: C, 36.53, H, 3.07, N, 10.55.

Additionally, the new complex has been studied using several spectroscopic methods, where the results are following:

*UV-Vis*: The regions of the maximum absorption occur at 409 nm and 548 nm (in DMSO).

*MALDI-TOF-MS*: m/z 394.0 (M)^+^, m/z 358.1 (M minus 2 H_2_O)

*IR*: 3090.5 cm^−1^ hydrogen bonds, 1660.8 cm^−1^ C=O, 1476.6 cm^−1^ C-C (aromatic) stretching vibrations, 822.4 cm^−1^ C-N (aromatic), 1607.1 cm^−1^ O-C=O, 769.7 cm^−1^ Cr-O.

^1^H NMR and ^13^C NMR spectra were not recorded due to the low solubility of [Cr(2-pic)_2_(OH_2_)_2_]NO_3_ in deuterated solvents.

### The oligomerization of 2-chloro-2-propen-1-ol

**Figure 3:**
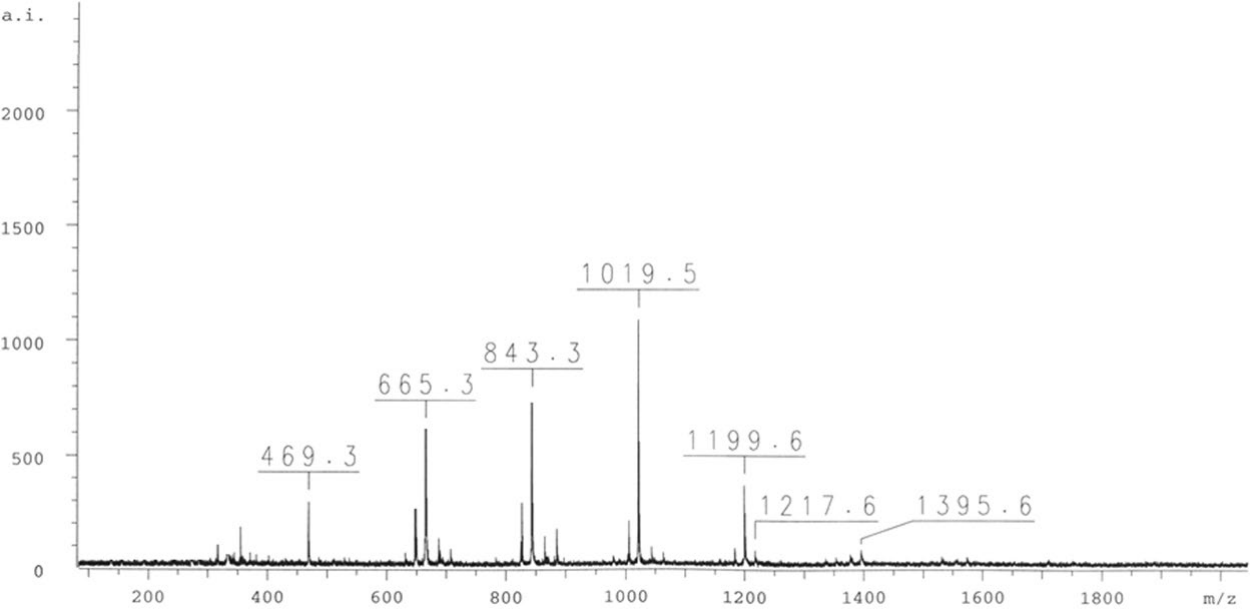
The MS spectrum for the oligomer consisting of 11 monomers (2-chloro-2-propen-1-ol).

**Figure 4:**
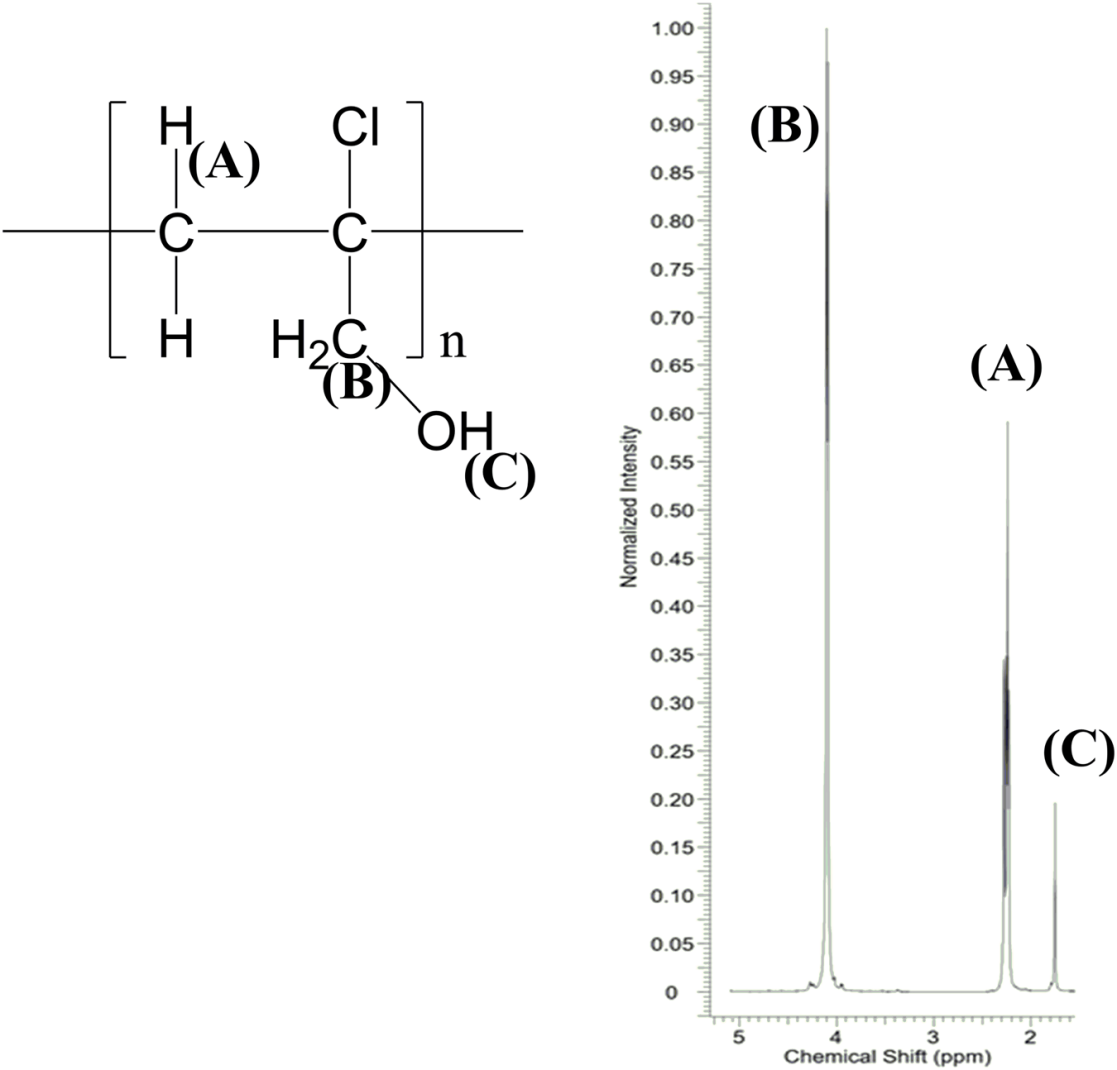
The ^1^H NMR spectrum for the system: the oligomer of 2-chloro-2-propen-1-ol (11 monomers), [Cr(2-pic)_2_(OH_2_)_2_]NO_3_ and MMAO-12.

**Figure 5:**
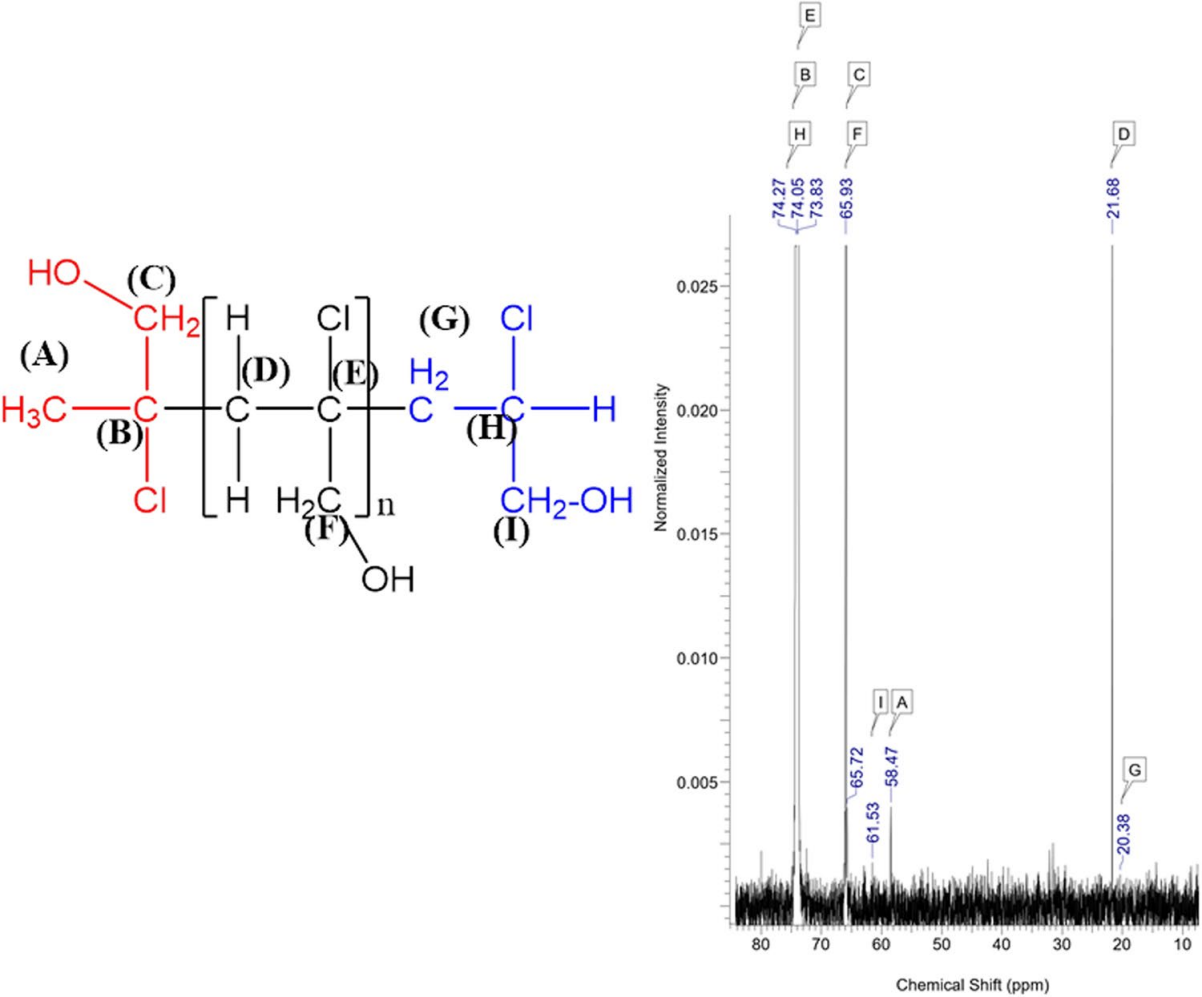
The ^13^C NMR spectrum for the system: the oligomer of 2-chloro-2-propen-1-ol (11monomers), [Cr(2-pic)_2_(OH_2_)_2_]NO_3_ and MMAO-12.

The new complex compound has been investigated as catalyst in the case of the 2-chloro-2-propen-1-ol oligomerization. After mixing monomers and activated [Cr(2-pic)_2_(OH_2_)_2_]NO_3_ (by MMAO-12) the oligomerization has been proceeded at the room temperature and at the atmospheric pressure. The poly-2-chloroallyl alcohol has been obtained as the product of the reaction. The oligomer of 2-chloro-2-propen-1-ol contains 11 monomers. The composition of the obtained oligomer has been confirmed by the spectroscopic methods including NMR and MS (Figs 3–5). Moreover, the catalytic activity of [Cr(2-pic)_2_(OH_2_)_2_]NO_3_ has been calculated. It equals 1434.33 g∙mmol^−1^∙h^−1^ for the molar ratio ${\rm{c}}{\rm{o}}{\rm{m}}{\rm{p}}{\rm{l}}{\rm{e}}{\rm{x}}[{\rm{C}}{\rm{r}}{(2-{\rm{p}}{\rm{i}}{\rm{c}})}_{2}{({{\rm{O}}{\rm{H}}}_{2})}_{2}]{{\rm{N}}{\rm{O}}}_{3}$: MMAO = 1: 1000.

## Discussion

The new complex - [Cr(2-pic)_2_(OH_2_)_2_]NO_3_ exhibits a very high catalytic activity for the oligomerization of 2-chloro-2-propen-1-ol. The catalysts with an activity higher than 1000 g∙mmol^−1^∙h^−1^ are assumed to be the very highly active catalysts^3^. Thus, it has been concluded that [Cr(2-pic)_2_(OH_2_)_2_]NO_3_ (1434.33 g∙mmol^−1^∙h^−1^) is a remarkably active catalyst. The analysis of the poly(2-chloroallyl alcohol) by mass spectrometry shows that the oligomer consisting of 11 monomers is formed. The molar mass of this oligomer is 1019.5 g∙mol^−1^ (Fig. 3). The MS spectrum of the oligomer shows that the peak of the highest intensity occurs at 1019.5 m/z. This value responds to 11 linked monomers of 2-chloro-2-propen-1-ol. Moreover, the distribution of mass peaks shows that the peaks differ about 185 m/z and this difference in the m/z value responds to the molecular weight of two monomers of 2-chloro-2-propen-1-ol. The MS spectrum confirms that the distribution of the obtained oligomer occurs every two molecules of 2-chloro-2-propen-1-ol. Figure 3 shows three oligomers: the first contains 5 monomers (469.3 m/z), the second contains 7 monomers (665.3 m/z) and the third oligomer about 9 monomers (843.3 m/z).

Furthermore, ^1^H and ^13^C NMR methods reveal the isotactic molecular structure of the obtained oligomer. A small number of signals in range 20 ppm – 75 ppm in the ^13^C NMR spectrum of the system: the oligomer of 2-chloro-2-propen-1-ol (11 monomers), [Cr(2-pic)_2_(OH_2_)_2_]NO_3_ and MMAO-12 confirms the isotactic structure of the obtained oligomer (Fig. 5)^16,17^. ^13^C NMR spectrum shows that there are three very high peaks at 74 ppm, 66 ppm and 22 ppm. Others peaks have a very low intensity. The configuration diversity visible on various carbon signals in ^13^C NMR makes it possible to propose the tacticity of the obtained oligomer.

Recently, the first example of the poly(2-chloroallyl alcohol) preparation catalyzed by the complex compounds was reported in the literature^15^. Two chromium(III) complex compounds containing both organic cations and anions, namely [Cr(dipic)_2_][Cr(bipy)(dipic)H_2_O]∙2H_2_O and [Cr(dipic)_2_]Hdmbipy∙2.5H_2_O were reported to exhibit a very high catalytic activity for the 2-chloro-2-propen-1-ol polymerization. These compounds have two times higher catalytic activity when compared to the catalyst described in this work - [Cr(2-pic)_2_(OH_2_)_2_]NO_3._ It may be explained by the fact that [Cr(dipic)_2_][Cr(bipy)(dipic)H_2_O]∙2H_2_O and [Cr(dipic)_2_]Hdmbipy∙2.5H_2_O complexes contain organic anions which may play an important role in interactions with MMAO in the polymerization mechanism.

In addition to the report referred above^15^, so far in the literature there are no reports on the oligomerization or polymerization of 2-chloro-2-propen-1-ol catalyzed by any complex compound with MMAO. Thus, in order to compare the catalytic activity of the new catalyst - [Cr(2-pic)_2_(OH_2_)_2_]NO_3_ with others catalysts known in the literature, we have collected the polymerization data for the selected chromium(III) complexes in Table 1. These complexes were selected for non-metallocene structure that they are as close as possible to the catalyst described in this work. As seen, [Cr(2-pic)_2_(OH_2_)_2_]NO_3_ as catalyst exhibits minimum about 1.4 and maximum 13.3 times higher catalytic activity than the catalysts compiled in Table 1.

**Table 1:** The collection of catalytic activities of non-metallocene chromium(III) complexes for the ethylene polymerization.

Complex	Temperature [°C]	Catalytic activity (g∙mmol^−1^∙h^−1^∙bar^−1^)	References
[Cr{2-[2-(diphenylphosphino)-1-(N-methylimidazol-2-yl) ethyl]-N-methy limidazole}Cl_3_]	100	108	^24^
[Cr{tris(*N*-methylimidazol-2-yl)methoxymethane}Cl_3_]	100	208	^24^
[Cr(1,3,5-triazacyclohexane)]Cl_3_	40	717	^25^
[(2,6-Me_2_Ph)_2_(nacnac)Cr (OEt_2_)CH_2_SiMe_3_] B(3,5-(CF_3_)_2_C_6_H_3_)_4_ (nacnac = 2,4-pentane-N,N’-bis(aryl)ketiminato)	75	228	^26^
CrMe[N(SiMe_2_CH_2_PPh_2_)_2_	300	500	^1^
[2,6-bis(imino)pyridyl]CrCl_3_	70	1000	^27^

## Conclusions

The composition and structure of the new complex catalyst- [Cr(2-pic)_2_(OH_2_)_2_]NO_3_ has been confirmed by several methods: NMR, MS, IR, UV-Vis, elemental analysis and the X-ray diffraction. The designed and synthesized [Cr(2-pic)_2_(OH_2_)_2_]NO_3_ exhibit the very high catalytic activity in the case of the 2-chloroallyl alcohol oligomerization. The oligomerization with the use of the new 2-pyridinecarboxylate complex of chromium(III) after the activation by MMAO-12 undergoes very easily at the room temperature and at atmospheric pressure. The product of the oligomerization reaction with the use of [Cr(2-pic)_2_(OH_2_)_2_]NO_3_ as catalyst is the poly(2-chloro-allyl alcohol) consisting of 11 monomers_._ The obtained oligomer has an isotactic structure.

The reported oligomerization results are promising. This means that the results described in this report give perspectives of the use of the new catalyst to the oligomerization of other beta-olefin derivatives. This kind of oligomers is used in the industrial production of elastomers and coatings.

## Methods

### Materials

The reagents were purchased from Sigma-Aldrich: 2-pyridinecarboxylic acid (2-pic), 99% purity), chromium(III) nitrate hexahydrate (99% purity), lithium carbonate (99% purity), toluene (99% purity), modified methylaluminoxane (MMAO-12, 7 wt% aluminum in toluene), 2-chloro-2-propen-1-ol (90%), and from Stanlab - nitric acid (65%).

### Synthesis

40 ml of the 0.7 M HNO_3_ solution has been mixed with Cr(NO_3_)_3_∙9H_2_O (10 mmol, 4.0 g) and 2-pyridinecarboxylic acid (2-pic) (22 mmol, 2.7 g). The reaction mixture has been heated at reflux for 30 minutes. Then the solution obtained by dissolving (8 mmol, 0.59 g) of Li_2_CO_3_ in 8 mL H_2_O was added to the reaction mixture. After the addition of Li_2_CO_3_ solution, the reaction mixture changed the color from green to purple. Then the solution has been heated for 5 hours at reflux. In the next step the reaction mixture has been cooled in a refrigerator. After cooling, the obtained product was filtered off and washed with water cooled to about 2 °C. To obtain the crystals of [Cr(2-pic)_2_(OH_2_)_2_]NO_3_, the powder was again dissolved in 0.1 M HNO_3_ (preheated to 100 °C). Next, the hot solution was filtered and left to cool. Then the red crystals of [Cr(2-pic)_2_(OH_2_)_2_]NO_3_ were obtained. The yield of the synthesis was 62%.

### X-Ray measurements

Good-quality single-crystal samples of [Cr(2-pic)_2_(OH_2_)_2_]NO_3_ were selected for the X-ray diffraction measurements (295(2) K) carried out on the Oxford Diffraction Gemini R ULTRA Ruby CCD diffractometer with the Mo *Kα* (λ = 0.71073 Å) radiation. The structure of [Cr(2-pic)_2_(OH_2_)_2_]NO_3_ was solved with the SHELX package and the SHELXL-97 program. CrysAlis CCD has been used to determine the lattice parameters^18,19^. The standard geometrical calculations linked with the crystal structure of the new complex were made with the PLATON program^20^. The following programs PLUTO-78, ORTEPII and Mercury were used to an analysis and a presentation of molecular structures^21–23^.

Full crystallographic details of [Cr(2-pic)_2_(OH_2_)_2_]NO_3_ have been deposited in the Cambridge Crystallographic Data Center (deposition No. CCDC 1811764) and they may be obtained from www: http://www.ccdc.cam.ac.uk, e-mail: deposit@ccdc.cam.ac.uk or The Director, CCDC, 12 Union Road, Cambridge, CB2 1EZ, UK.

### IR spectra

The IR spectrum (4000-650 cm^−1^ range) were obtained using the BRUKER IFS 66 spectrophotometer over the in a KBr pellet.

### UV-Vis spectra

The UV-Vis spectrum were registered on the Perkin-Elmer Lambda 650. The instrument is linked with the temperature control system (with a scan accuracy of 1 nm and a 1 nm slit width at a scanning rate 120.00 nm min^−1^ (298 K) - Peltier System. The spectrum of [Cr(2-pic)_2_(OH_2_)_2_]NO_3_ was recorded for the solution of this complex in DMSO (C_complex_ = 5 mM).

### MS spectra

The positive-ion mode MALDI-TOF mass spectrum were obtained using the Bruker Biflex III spectrometer. 2,5-Dihydroxybenzoic acid (DHB) was used as a matrix.

### NMR spectra

The ^1^H and ^13^C NMR spectra of the system: the oligomer of 2-chloro-2-propen-1-ol (11monomers), [Cr(2-pic)_2_(OH_2_)_2_]NO_3_ and MMAO-12 were recorded on the Bruker Avance III 500 (500.13/125.76 MHz) instrument (300 K). The poly(2-chloroallyl alcohol) was dissolved in C_2_D_2_Cl_4_.

### The oligomerization process

The oligomerization experiments were carried out at atmospheric pressure and at 21 °C under the nitrogen atmosphere. The red solution of [Cr(2-pic)_2_(OH_2_)_2_]NO_3_ (3 μmol, 1.2 mg) in toluene (2 mL) was placed using a glass syringe in the glass cell with a sealed stopper. The glass cell was placed on a magnetic stirrer throughout the duration of the experiments. In the next step, MMAO-12 solution (3 mL) was added to the toluenic solution of the new chromium(III) complex. After the addition of the MMAO-12 solution the reaction mixture changed color to brown. 2-chloro-2-propen-1-ol as monomer (3 mL) was added to the glass cell with MMAO-12 and the solution of chromium(III) complex. The oligomerization reaction was carried out for 45 minutes. After this time the sticky gel was obtained. The sample of the obtained oligomer, poly(2-chloroallyl alcohol), has been weighed. The product of the oligomerization has been characterized by the positive-ion mode MALDI-TOF mass spectrometry throughout selecting a matrix that facilitates its ionization (DHB). The MALDI-TOF was used to the direct molecular weight determination of the oligomeric poly(2-chloroallyl alcohol). Moreover, the oligomer has been examined by the NMR spectroscopy. The sample of the oligomer in a small vial was dissolved in 1,1,2,2-tetrachloro(^2^H_2_)ethane. Next, it was transferred using a glass Pasteur pipette to the NMR tube. The analysis of the NMR spectra has been conducted on the ACD/NMR Processor Academic Edition computer program.

## Electronic supplementary material

Crystallography data
