# Supplementary material for: Oligomerization of 2-chloroallyl alcohol by 2-pyridinecarboxylate complex of chromium(III) - new highly active and selective catalyst
Source: Sci Rep. 2018 Jun 5;8:8632. doi: 10.1038/s41598-018-26973-6 (PMC5988823; doi:10.1038/s41598-018-26973-6)
Supplement: Supplementary file 1 — Crystallography data [file 41598_2018_26973_MOESM1_ESM.pdf]

Oligomerization of 2-chloroallyl alcohol by 2-pyridinecarboxylate complex of chromium(III) - new highly active and selective catalyst

Joanna Drzeżdżon*, Artur Sikorski, Lech Chmurzyński, Dagmara Jacewicz

Table 1. Crystallographic data for $[Cr(2\text{-pic})_2(H_2O)_2]NO_3$

Chemical formula	$C_{12}H_{12}CrN_3O_9$
FW/g mol^{-1}	394.25
Crystal system	Monoclinic
Space group	*C*2/c
a/Å	10.4498(5)
b/Å	9.4951(4)
c/Å	15.9025(7)
β/°	108.319(5)
V/Å^3	1497.90(11)
Z	4
T/K	295(2)
ρ_{calc}/g cm^{-3}	1.748
F(000)	804
μ/mm^{-1}	0.822
2θ range for data collection/º	3.52 - 25.00
Completeness 2θ /%	99.8
Reflections collected	4541
Reflections unique	1321 [$R_{(int)}$ = 0.0294]
Data/restraints/parameters	1321 / 2 / 122
Goodness-of-fit on F^2	1.070
Final R_1 value ($I > 2\sigma(I)$)	0.0295
Final wR_2 value ($I > 2\sigma(I)$)	0.0764
Final R_1 value (all data)	0.0343
Final wR_2 value (all data)	0.0787
CCDC number	1811764

Table 2. Selected bond lengths (Å) and angles (°) in title compounds.

Bond lengths	(Å)	Valence angles	(°)	Torsion angles	(°)
Cr1-O1	1.9452(13)	O1^i-Cr1-O1	180.00(8)	O1-Cr1-N1-C6	179.8(2)
Cr1-O1W	1.9938(17)	O1^i-Cr1-O1W	89.20(6)	O1W^i-Cr1-N1-C6	90.9(2)
Cr1-N1	2.0368(17)	O1-Cr1-O1W	90.80(6)	O1W-Cr1-N1-C2	89.67(15)
Cr1-O1^i	1.9452(13)	O1^i-Cr1-N1	99.07(6)	N1^i-Cr1-N1-C6	93(100)
Cr1-O1W^i	1.9938(17)	O1-Cr1-N1	80.93(6)	C6-N1-C2-C7	-178.84(19)
Cr1-N1^i	2.0368(17)	O1W-Cr1-N1	88.72(7)	O1-Cr1-N1-C2	-90.33(15)

Table 3. Hydrogen bonding interactions in the crystal structure of [Cr([Cr(2-pic)$_2$(OH$_2$)$_2$]NO$_3$.

D–H···A	*d*(D–H) (Å)	*d*(H···A) (Å)	*d*(D···A) (Å)	<D–H···A (°)
O1W–H1WA···O2^{iii}	0.82(3)	1.88(3)	2.703(2)	177(3)
O1W–H1WB···O3	0.81(3)	2.46(2)	3.057(2)	132(2)
O1W–H1WB···O4	0.81(3)	1.94(3)	2.738(2)	171(3)
Symmetry codes: (iii) ½+x, –½+y, z.				
